# Post-surgical thyroid bed myofibroma simulating a recurrent papillary thyroid carcinoma: A case report and review of the literature

**DOI:** 10.1097/MD.0000000000036945

**Published:** 2024-01-12

**Authors:** Jun Hyeon Park, Kyung Sik Yi, Chi-Hoon Choi, Yook Kim, Jisun Lee, Yeongtae Park, Ok-Jun Lee

**Affiliations:** a Department of Radiology, Chungbuk National University Hospital, Cheongju, Republic of Korea; b College of Medicine and Medical Research Institute, Chungbuk National University, Cheongju, Republic of Korea; c Department of Pathology, Chungbuk National University Hospital, Cheongju, Republic of Korea.

**Keywords:** core needle biopsy, fine needle aspiration, myofibroma, post-surgical thyroid bed, recurrence

## Abstract

**Rationale:**

Myofibromas are rare benign spindle cell tumors of the soft tissue, bone, or internal organs that occur at any age. Here, we report a post-surgical thyroid bed myofibroma that mimicked a papillary thyroid carcinoma.

**Patient concerns:**

A 56-year-old male presented with a mass in the thyroid surgical bed, detected 3 years post thyroidectomy following papillary carcinoma.

**Diagnosis:**

Thyroid ultrasonography revealed a well-defined, lobulated, hypoechoic, solid nodule, with large rod-like echogenic foci in the thyroid surgical bed. The development of a postoperative suture granuloma was considered. However, ultrasonography performed 12 months later showed a marked increase in the lesion size. Two fine needle aspiration cytology yielded nondiagnostic results.

**Intervention:**

Considering the possibility of local tumor recurrence, surgical resection was performed.

**Outcome:**

The diagnosis of a myofibroma was confirmed, and no additional treatment was administered.

**Lessons:**

It is challenging to differentiate lesions occurring on the thyroid surgical bed after surgery, from recurrent thyroid cancer. A lesion measuring 6 mm, with a degree of punctate echogenicity, suggests tumor recurrence. Moreover, myofibromas are extremely rare. This case highlights that it is advisable to perform a core needle biopsy in cases of nondiagnostic fine needle aspiration results.

## 1. Introduction

Myofibromas are rare benign spindle cell tumors composed of myofibroblasts that can occur in any age group. Infantile myofibromatosis, typically detected as solitary or multiple masses, commonly develop in those less than 2 years old.^[1,2]^ In adults, myofibromas usually present as solitary superficial masses in any part of the body.^[3]^ We report a post-surgical thyroid bed myofibroma that mimicked a recurrent papillary thyroid carcinoma. In our case, the tumor size increased significantly during the follow-up period and appeared to be in contact with the trachea, leading to the misdiagnosis of a local tumor recurrence of his thyroid cancer.

## 2. Case report

### 2.1. Patient information and clinical findings

**Figure 1.**
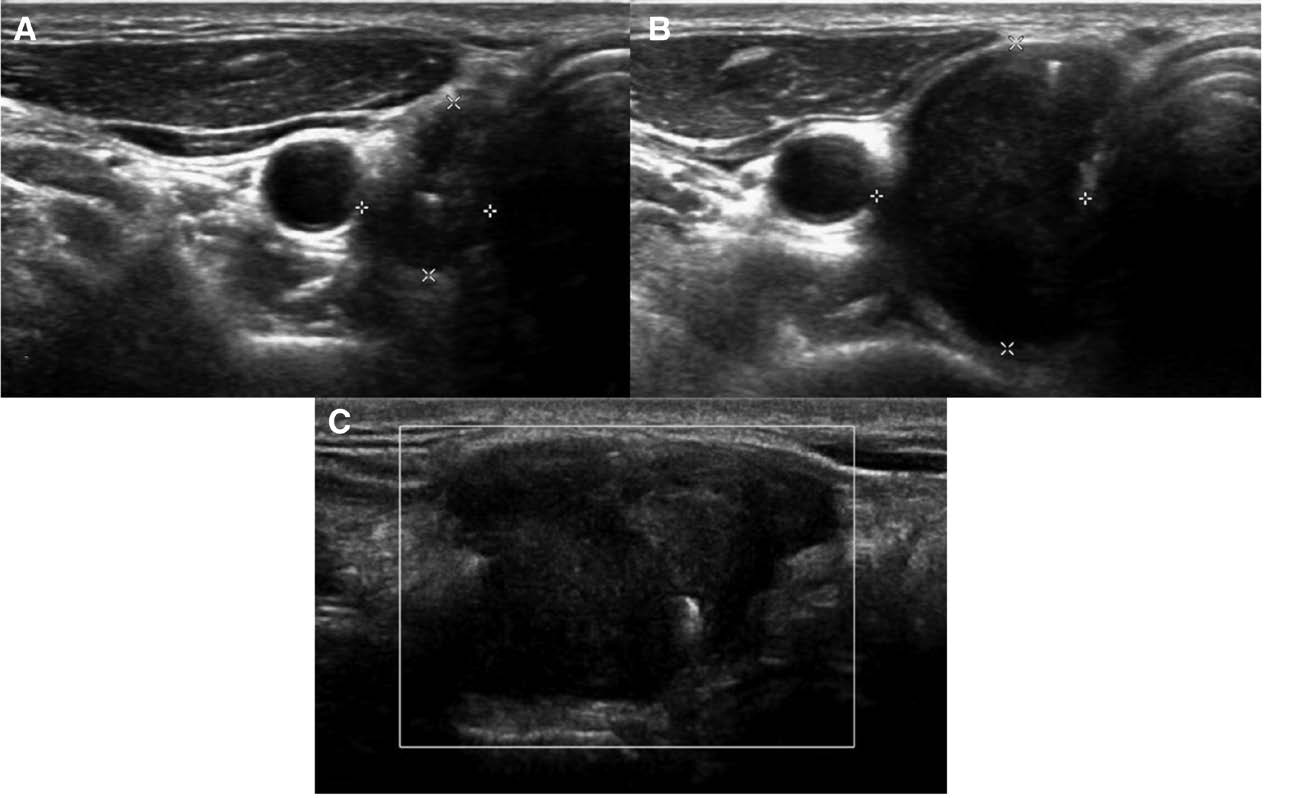
A 56-year-old male presented a thyroid surgical bed mass, detected via ultrasonography, 3 years after a total thyroidectomy for a papillary carcinoma. (A) Transverse ultrasound images revealed a 1.0 × 1.3 × 2.5 cm, irregularly-shaped, marked, hypoechoic lesion, with probable internal surgical clips. Ultrasound performed 12 months after the initial ultrasound. (B) Transverse ultrasound images showed a marked increase in the lesion size. (C) Doppler image showed no increased vascularity in the lesion.

A 56-year-old male presented with a mass in the thyroid surgical bed, detected via ultrasonography, 2 years post thyroidectomy for a papillary carcinoma. His thyroid function tests were normal, and he was asymptomatic. Ultrasonography revealed a well-defined, lobulated, hypoechoic, solid nodule, with large rod-like echogenic foci (Fig. 1A). The development of a postoperative suture granuloma was considered. However, ultrasonography performed 12 months later showed a marked increase in the lesion size (Fig. 1B and C). Because of the increased size of the lesion, the development of a recurrent papillary carcinoma was highly suspected.

Ultrasound-guided fine-needle aspiration (FNA) yielded nondiagnostic results. A repeat FNA was performed, as recommended by treatment guidelines,^[4]^ however the results were still nondiagnostic.

### 2.2. Therapeutic interventions

**Figure 2.**
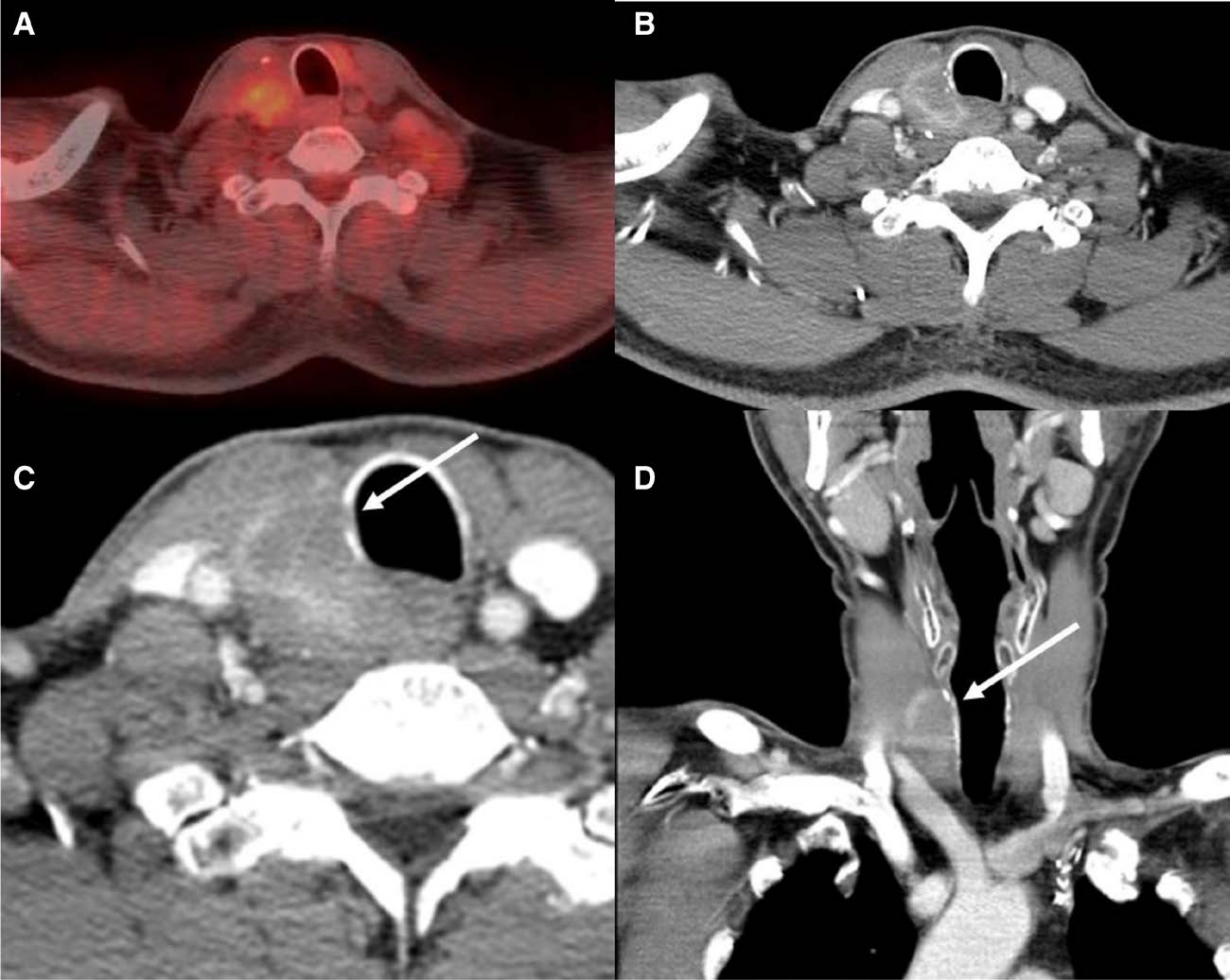
Preoperative imaging studies. (A) On 18^F^-fluorodeoxyglucose positron emission tomography-computed tomography, a right thyroid operative bed lesion showed heterogeneous hypermetabolism. (B) An axial computed tomography image showed an ill-defined hypo-enhancing mass at the right thyroid operative bed. (C) Axial and (D) coronal images showed focal decreased density at the right tracheal cartilage (arrow).

Surgery was performed under the impression of a possible local tumor recurrence. Pre-operative workup, including contrast-enhanced neck computed tomography (CT) and positron emission tomography-CT (PET-CT), were performed. A heterogeneous hypermetabolic mass detected on PET-CT (Fig. 2A). Enhanced neck CT showed an ill-defined hypo-enhancing mass at right thyroid surgical bed (Fig. 2B), and a focal defect on the right side of the tracheal cartilage (Fig. 2C and D), suggested possible local tumor recurrence and invasion to the trachea.

Excision of the neck mass was performed. A frozen biopsy of the resected specimen revealed a benign soft tissue. The patient was in a stable condition postoperative, and his thyroid function tests remained unremarkable.

### 2.3. Pathologic findings

**Figure 3.**
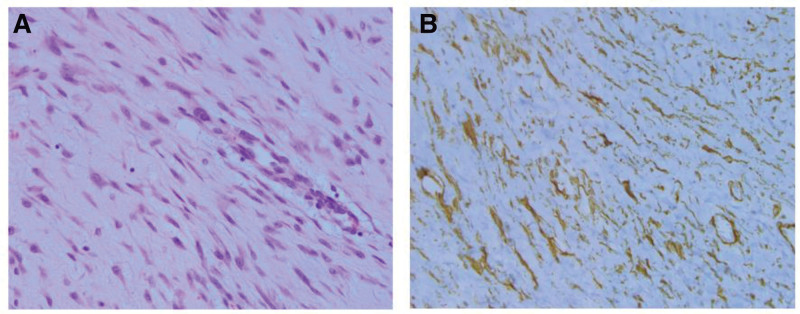
Histopathological examination of the surgical specimen. (A) Hematoxylin and eosin staining (×400), Specimen consisting of bland-looking spindle cells. (B) Immunohistochemical examination (SMA), the spindle cells expressed smooth muscle actin.

The tumor was well-demarcated and consisted of spindle cells in a fibromyxoid background. The nuclei were elongated and tapered, and lacked nuclear atypia (Fig. 3A). Immunohistochemistry examination was performed to identify the nature of the spindle cells. The tumor cells stained focally for smooth muscle actin and did not express CD34, S100, or desmin (Fig. 3B). Based on the tumor microscopic and immunohistochemical features, this spindle cell tumor was consistent with a myofibroma.

### 2.4. Ethical considerations

The institution review board approved the retrospective review of the medical records of this patient, and waived the requirement to obtain informed consent.

## 3. Discussion

Myofibromas are rare benign spindle cell tumors composed of myofibroblasts that can occur in any age group. This type of tumor is pathologically distinct from postoperative granulomas, which commonly occur in the surgical bed. Postoperative granulomas are characterized by myxoid substances and inflammatory cells.^[5]^ Although some case reports have mentioned the occurrence of benign tumors after thyroidectomy, there are only a few reports on spindle cell tumors.^[6,7]^

Ultrasonography is the primary imaging modality for the detection of postoperative lesions and assessment of recurrent malignancies. Thyroid bed lesions are commonly seen on ultrasonography, and can occur in up to 40% of postoperative cases.^[8]^ For effective management, it is essential to distinguish postoperative lesions from local tumor recurrences. Unfortunately, sonographic features, including cystic components, echogenicity, fatty hilum, and the tumor shape, are insufficient to differentiate local tumor recurrence. Only a lesion size of 6 mm or larger and the presence of punctate echogenic foci are considered significant indicators of local tumor recurrence.^[8–12]^ Thus, when evaluating for possible tumor recurrence, it is crucial to consider not only the lesion sonographic features but also other clinical findings.

In particular, an increase in serum thyroglobulin (Tg) levels during serial follow-ups has been reported to significantly predict tumor recurrence.^[13]^ However, some reports state the difficulty of predicting tumor recurrence based solely on serum Tg levels.^[14]^ Thus, a comprehensive assessment that considers both sonographic features and serum Tg levels is necessary. In this case, the patient postoperative serum Tg level was < 0.2 ng/mL. Three years later, his Tg level increased to 2.15 ng/mL. Ultrasonography revealed a lesion measuring approximately 2.5 cm. Therefore, considering the criteria for sonographic features indicative of recurrence, particularly focusing on the lesion size and the accompanying slight elevation in serum Tg levels, local tumor recurrence was suspected.

FNA is a readily available and accurate method for evaluating head and neck masses. However, in the case of thyroid bed lesions, FNA may yield inconclusive results due to low cellularity or non-representative sampling.^[15]^ Studies have reported a wide range (50%–95%) of conclusive results for FNAs of thyroidectomy bed lesions.^[8,12,16,17]^ In our case, a repeat FNA was performed because of the initial non-diagnostic result, but it failed to provide a conclusive diagnosis. Recently, ultrasound-guided core needle biopsy (CNB) has been suggested as an adjunct to FNA, and has been reported to be accurate and safe for evaluating thyroidectomy bed lesions.^[15]^ Considering the size of our patient lesion, which was larger than 2 cm, CNB could have been used as a complementary diagnostic tool.

## 4. Conclusion

Post-surgical thyroid bed myofibroma is challenging to differentiate from local tumor recurrence due to its rarity. Thus, in cases of thyroid bed nodules with nondiagnostic FNA results, it may be helpful to perform an additional CNB.

## Author contributions

**Supervision:** Chi-Hoon Choi, Jisun Lee, Yook Kim, Yeongtae Park, Ok-Jun Lee.

**Writing – original draft:** Jun Hyeon Park.

**Writing – review & editing:** Kyung Sik Yi.
